# Ribosome engineering enhances genetic code expansion in *Saccharomyces cerevisiae*

**DOI:** 10.1016/j.synbio.2026.08.009

**Published:** 2026-08-29

**Authors:** Xiaoxu Chen, Wenlu Shen, Xianqing Chen, Fangfang Zhang, Bo Yang, Zhiyong Yue, Guanghou Zhao

**Affiliations:** a Key Laboratory of Natural Anti-aging Product Mining and Biosynthesis of Shaanxi Higher Education Institutes, College of Medicine, Xi’an International University, Xi’an, 710077, China; b School of Life Science and Technology, Northwestern Polytechnical University, Xi’an, Shaanxi, 710129, China; c Biocytogen (Beijing) Co., Ltd., Beijing, 102609, China

**Keywords:** Genetic code expansion, Ribosome engineering, Noncanonical amino acid, *Saccharomyces cerevisiae*

## Abstract

Genetic code expansion enables the site-specific installation of noncanonical amino acids (ncAAs) into proteins, but its limited efficiency in eukaryotes remains a major barrier to broader application. Here we establish a visual, plug-and-play screening platform to evolve 18S ribosomal DNA in *Saccharomyces cerevisiae* and identify ribosomal variants that improve ncAA incorporation. The best-performing strain, designated ribo-hyper, increased ncAA-dependent GFP production by 2.9-fold relative to the wild-type rDNA strain and enhanced incorporation across distinct orthogonal aminoacyl-tRNA synthetase/tRNA pairs. Characterization of ribo-hyper showed that global translation activity and cellular growth were moderately reduced. Proteomic analysis further revealed changes in amino acid biosynthesis, translation-related proteins and stress-response pathways, indicating that the engineered ribosome reshapes cellular translation homeostasis. Perturbation of translation quality-control pathways, including the ribosome-rescue factors Dom34 and Hbs1 and the core mRNA exosome component Ski6, reduced ncAA-containing protein output, whereas disruption of ribosome quality-control factor Rqc2 had little effect. These findings support a role for ribosome rescue and associated mRNA turnover in efficient ncAA incorporation in the ribo-hyper strain. Together, our results establish eukaryotic ribosome engineering as a viable strategy for improving genetic code expansion in yeast.

## Graphical abstract

**Figure:**
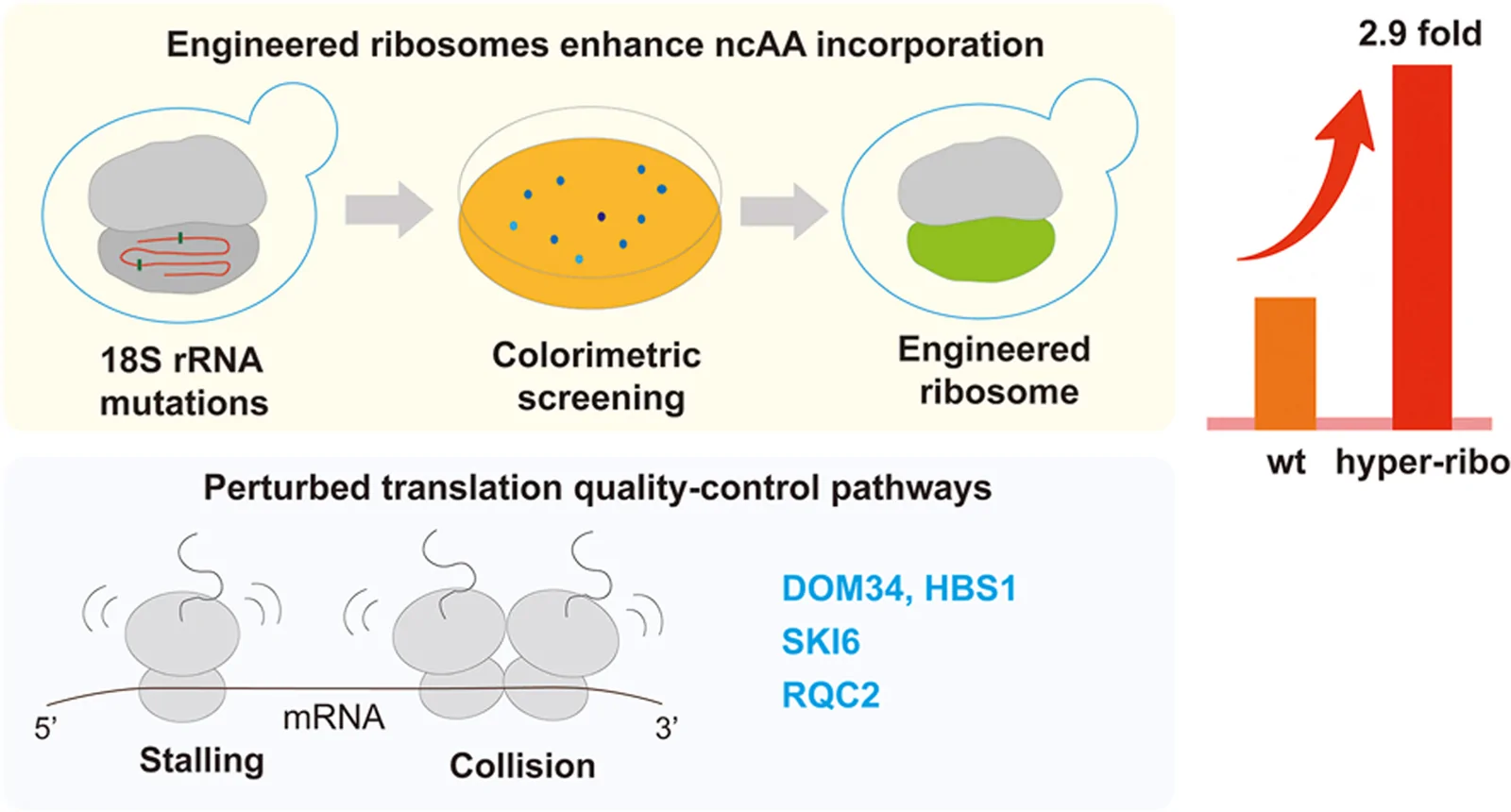

## 1 Introduction

**Fig. 1:**
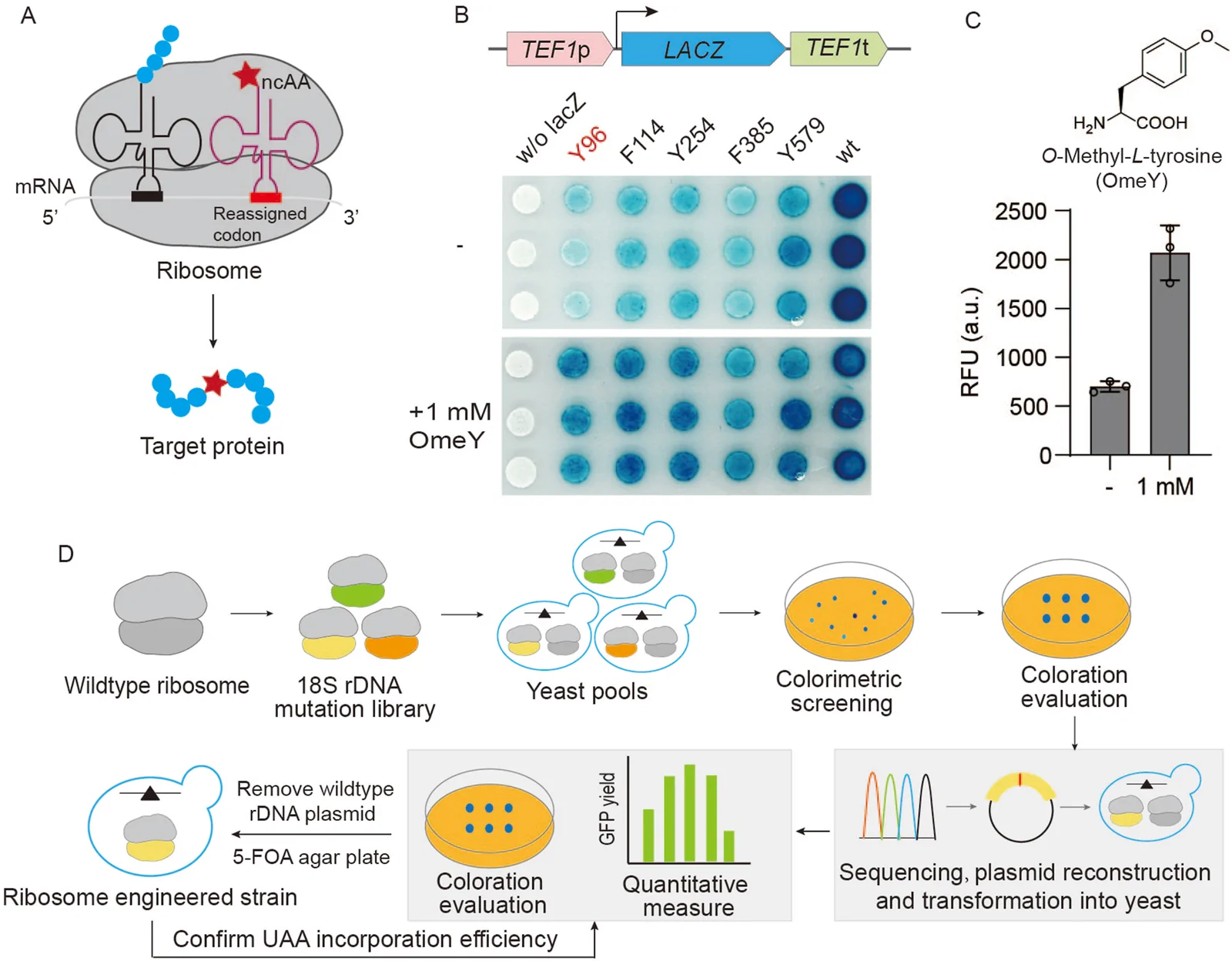
Design of the colorimetric selection system and screening workflow for yeast 18S rDNA mutagenesis libraries. (A) Schematic of site-specific ncAA incorporation on the ribosome. An orthogonal tRNA (red color) charged with an ncAA (red star) recognizes the reassigned codon (red color) in the mRNA and directs ncAA incorporation into the nascent polypeptide. (B) Evaluation of LacZ(TAG) reporters for colorimetric selection. Individual LacZ residues were replaced with TAG codons to couple β-galactosidase activity to ncAA incorporation. LacZ variants were expressed from a single-copy plasmid and assayed in the absence or presence of 1 mM OmeY. w/o, strain lacking the LacZ expression cassette; WT, wild-type LacZ. LacZ(Y96TAG) was selected for high-throughput mutagenesis screening. Data are from three biological replicates. (C) Quantification of ncAA incorporation using a GFP reporter. Relative GFP fluorescence was measured in the absence or presence of 1 mM OmeY and normalized to cell density. (D) Workflow for colorimetric screening of yeast 18S rDNA mutagenesis libraries.

Genetic code expansion (GCE) enables the site-specific, co-translational incorporation of noncanonical amino acids (ncAAs) into proteins through bio-orthogonal aminoacyl-tRNA synthetase (aaRS)/tRNA pairs [1,2] (Fig. 1A). Hundreds of ncAAs bearing diverse chemical, physical and biological functionalities have now been genetically encoded, supporting applications in protein engineering, chemical biology, therapeutics, biocatalysis and bioremediation [3,4]. Despite this progress, inefficient ncAA incorporation remains a central limitation, particularly in eukaryotic cells.

Most efforts to improve GCE have focused on the orthogonal translation components that directly charge and decode the ncAA, including engineered aaRSs and suppressor tRNAs [[5], [6], [7], [8], [9], [10], [11]]. Host translation factors also shape ncAA incorporation efficiency, as demonstrated by strategies that modify elongation factors [[12], [13], [14]] or release factors to improve suppression of reassigned codons [[15], [16], [17], [18]]. These studies indicate that ncAA incorporation is not determined solely by the orthogonal aaRS/tRNA pair, but also by the properties of the host translational apparatus.

The ribosome is therefore an attractive but challenging target for improving GCE. Ribosome engineering has advanced substantially in bacteria, where orthogonal ribosomes, in vitro selection systems and high-throughput screens have enabled variants with improved genetic code expansion properties [[19], [20], [21], [22], [23], [24], [25], [26]]. In eukaryotes, however, ribosome engineering remains much less developed. Eukaryotic rRNAs are more complex, ribosome biogenesis is tightly linked to viability, and robust orthogonal or in vitro ribosome evolution platforms are not yet generally available. Whether engineering the eukaryotic ribosome can improve ncAA incorporation in vivo has therefore remained largely unexplored.

Beyond engineering the translational machinery itself, mRNA and protein surveillance pathways may also shape GCE efficiency. In yeast, deletion of the nonsense-mediated decay factor UPF1 increases the yield of ncAA-containing proteins [11], suggesting that reassigned stop codons can engage endogenous quality-control pathways. More broadly, translation-associated quality-control pathways monitor aberrant translation events and can promote mRNA degradation, clearance of incomplete nascent chains and ribosome recycling [[27], [28], [29], [30]]. During GCE, ncAA incorporation imposes several noncanonical features on translation, including competition between stop-codon suppression and termination, decoding by orthogonal tRNAs and, in some cases, accommodation of amino acid substrates that differ from canonical amino acids in size, geometry or chemical properties. These features may create translational contexts that are sensed by surveillance pathways, but whether such pathways influence ncAA-containing protein production remains unclear.

Here we developed a visual, plug-and-play selection platform for GCE-dependent screening in *S. cerevisiae*. Using this approach, we evolved 18S rDNA and identified ribosomal variants that increase ncAA incorporation, including a strain termed ribo-hyper that improved ncAA-dependent protein production by 2.9-fold. We further characterized the translation properties of ribo-hyper and examined how selected translation-associated quality-control factors affect ncAA-containing protein output. Together, our results support eukaryotic ribosome engineering as a practical strategy for improving genetic code expansion and suggest that ribosome rescue and mRNA turnover pathways can modulate the efficiency of ncAA-containing protein synthesis.

## 2 Materials and methods

### 2.1 Strains and plasmid construction

The strains, plasmids and primers used in this study are listed in Supplementary Tables S2–S4. *Saccharomyces cerevisiae* BY4741 (MATa *his3*Δ0 *leu2*Δ0 *met15*Δ0 *ura3*Δ0) was used as the parental strain. In a previous study from our laboratory, the entire chromosomal rDNA cluster of BY4741 was deleted, and cell viability was maintained by introducing a multicopy plasmid carrying a wild-type rDNA unit, pRDNwt-U (*URA3*). Homozygous 18S rDNA mutant strains were generated by replacing the wild-type rDNA plasmid with mutant rDNA plasmids, followed by counterselection of pRDNwt-U on 5-fluoroorotic acid (5-FOA) agar plates.

Deletion of genes involved in translation-associated quality-control pathways was performed using CRISPR-Cas9-mediated genome editing with donor DNA fragments containing homologous arms. Plasmids encoding evolved aminoacyl-tRNA synthetase/tRNA pairs were kindly provided by the Liu laboratory. The codon-optimized GFP gene (*yeGFP*) containing an amber stop codon was placed under the control of the *TEF1* promoter and *CYC1* terminator and inserted into these plasmids by Gibson assembly. Four copies of the tRNA cassette were generated through four sequential rounds of restriction-enzyme digestion and ligation. The amber-mutant LacZ reporter was generated by overlap-extension PCR. The LacZ reporter was then placed under the control of the *TEF1* promoter and *TEF1* terminator and inserted into a single-copy plasmid.

### 2.2 Construction of the 18S rDNA mutant plasmid library

The 18S rDNA mutant library was generated by error-prone PCR as previously described, with minor modifications [31]. Briefly, 18S rDNA was amplified using the low-fidelity EasyTaq DNA polymerase (TransGen Biotech, Beijing, China). Mutagenesis was promoted by adding the nucleotide analogues dPTP and 8-oxo-dGTP (Biorbyt, United Kingdom). The first-round PCR reaction was performed in a total volume of 20 μL containing 1.6 μL of 2.5 mM dNTPs, 0.4 μL of 10 mM dPTP, 0.4 μL of 10 mM 8-oxo-dGTP, 0.2 μL of EasyTaq DNA polymerase and 100 ng of template plasmid. PCR was performed according to the manufacturer’s instructions for four amplification cycles.

The PCR products were purified and digested with DpnI to remove the template plasmid. After purification, 1 μL of the product was used as the template for the second round of PCR amplification using Phanta Super-Fidelity DNA Polymerase (Vazyme, Nanjing, China) according to the manufacturer’s protocol. The resulting PCR products were purified, digested overnight with BsmBI and ligated into the digested recipient vector.

The recipient vector, which contained the ordered rDNA subset excluding the 18S rDNA sequence, was generated by Golden Gate assembly as previously described [32]. In this vector, the 18S rDNA region was replaced by an *RFP* cassette to facilitate plasmid construction. For library construction, the BsmBI-digested vector and BsmBI-digested 18S rDNA mutant fragments were ligated using T4 DNA ligase (Thermo Fisher Scientific) for 2 h at room temperature. The ligation products were transformed into XL10-Gold chemically competent cells. Transformants were plated on LB agar containing ampicillin and incubated at 30 °C until colonies reached the desired size. All colonies were pooled by washing the plates, and plasmids were extracted to obtain the 18S rDNA mutant plasmid library. This error-prone PCR strategy was expected to generate up to approximately ten mutations across the 1.8-kb 18S rDNA sequence.

### 2.3 Yeast media and growth conditions

Yeast strains were grown in YPD medium or synthetic dropout (SD) medium. YPD medium contained 2% glucose, 2% peptone and 1% yeast extract. SD medium contained 0.67% yeast nitrogen base and 2% glucose and was supplemented with the required amino acids. Solid media were prepared by adding 1.5% agar, and the pH was adjusted to 6.5 when required. Yeast strains were cultured at 30 °C.

### 2.4 Colorimetric plate-overlay assay

The colorimetric assay was performed using a plate-overlay method as previously described [33]. Briefly, yeast colonies grown on plates were lysed by gently flooding the plate surface with 2 mL of CHCl_3_ and allowing it to evaporate. This step was repeated twice. The plates were then overlaid with 10 mL of 1% agarose prepared in 100 mM Na_2_HPO_4_ buffer (pH 7.0) containing 250 mg/L X-gal. Plates were kept level until the agarose layer solidified and were then incubated at 37 °C for 12 or 24 h until blue color developed in colonies or spotted cells.

### 2.5 GFP fluorescence assay

Overnight yeast cultures were diluted into fresh medium to an initial OD600 of 0.1 and cultured at 30 °C with shaking at 220 rpm for 24 or 36 h. A total of 100 μL of culture was collected, washed with double-distilled water and resuspended in 100 μL of double-distilled water. Next, 40 μL of the cell suspension was mixed with 160 μL of double-distilled water in a 96-well plate. Fluorescence intensity was measured at an excitation wavelength of 485 nm and an emission wavelength of 528 nm, and OD600 was recorded using a Synergy Neo2 Multi-Mode Reader (BioTek). ncAA incorporation efficiency was calculated as fluorescence intensity normalized to OD600.

### 2.6 Dual-luciferase assay

Overnight yeast cultures were diluted into fresh medium to an initial OD600 of 0.1 and grown to mid-log phase (OD600 = 0.7–0.9). Cells equivalent to 0.5 OD600 units were collected and washed with double-distilled water. Cell pellets were lysed in 100 μL of Passive Lysis Buffer (Promega). Luminescence was measured using the Dual-Luciferase Reporter Assay System (Promega) according to the manufacturer’s instructions on a Synergy Neo2 Multi-Mode Reader (BioTek). For reporters carried on multicopy plasmids, 2 μL of cell lysate was used; for reporters carried on single-copy plasmids, 8 μL of cell lysate was used. Reporter activity was calculated as the ratio of Firefly luciferase activity to Renilla luciferase activity. Each assay was performed using four independent biological replicates.

### 2.7 Serial dilution assay

Single colonies were cultured overnight in liquid YPD medium at 30 °C. Cell density was adjusted to an OD600 of 1.0, and cultures were serially diluted tenfold in sterile water. Diluted cell suspensions were spotted onto the indicated plates and incubated at 30 °C for the indicated number of days before imaging.

### 2.8 Puromycin incorporation assay and western blotting

For puromycin incorporation analysis, overnight cultures were diluted into 5 mL of fresh YPD medium to an initial OD600 of 0.1. When cells reached mid-log phase (OD600 = 0.6–0.7), puromycin was added to a final concentration of 2 mM, and cultures were incubated for 1 h at 30 °C. Cells equivalent to 2 OD600 units were collected and washed with 400 μL of ice-cold double-distilled water. After centrifugation, cell pellets were resuspended in 100 μL of 20 mM NaOH and incubated for 5 min at room temperature. Samples were centrifuged again, and the supernatant was discarded. The pellets were resuspended in SDS sample buffer containing 60 mM Tris-HCl (pH 6.8), 10% glycerol, 2% SDS, 0.01% bromophenol blue, 0.01 M DTT and 5% β-mercaptoethanol, followed by heating at 100 °C for 10 min. Protein samples were collected from the supernatant after centrifugation.

A total of 5 μL of each protein sample was separated by 10% SDS-PAGE and analyzed by western blotting. Puromycin-incorporated nascent polypeptides were detected using an anti-puromycin monoclonal antibody (Merck, MABE343). GAPDH was used as the loading control and detected using an anti-GAPDH antibody (ABclonal, AC033).

### 2.9 Protein purification and mass spectrometry sample preparation

A single colony harboring a plasmid encoding C-terminal His6-tagged *yeGFP* (Y40TAG) and evolved *EcOmeYRS* was inoculated into 20 mL of liquid SD medium and cultured overnight. The saturated culture was diluted into 500 mL of fresh SD medium to an initial OD600 of 0.1 and supplemented with 5 mM OmeY. Cells were harvested after 36 h of shaking at 30 °C.

Cell pellets were resuspended in lysis buffer containing 10 mM imidazole, 300 mM NaCl and 50 mM NaH_2_PO_4_ (pH 8.0), supplemented with 1× protease inhibitor cocktail. Cells were lysed by ultrahigh-pressure homogenization at 1200 bar and 4 °C for 15 min. The lysate was clarified by centrifugation at 8000 rpm and 4 °C for 40 min. The supernatant was incubated with 1 mL of Ni-NTA agarose (Takara Bio) for 2 h at 4 °C with shaking. The resin was washed with five column volumes of wash buffer containing 20 mM imidazole, 300 mM NaCl and 50 mM NaH_2_PO_4_ (pH 8.0). His6-tagged proteins were eluted with elution buffer containing 250 mM imidazole, 300 mM NaCl and 50 mM NaH_2_PO_4_ (pH 8.0). Purified proteins were analyzed by 10% SDS-PAGE, and the approximately 27-kDa protein band was excised for high-resolution mass spectrometry analysis.

### 2.10 Sample preparation for proteomic analysis

Single colonies were inoculated into 5 mL of YPD medium and cultured overnight. Overnight cultures were diluted into 40 mL of fresh YPD medium to an initial OD600 of 0.1. Cells were harvested at mid-log phase (OD600 = 0.6–0.8), washed twice with ice-cold double-distilled water and immediately snap-frozen in liquid nitrogen. Three independent biological replicates were prepared for proteomic analysis. Detailed experimental and computational procedures for the TMT-based quantitative proteomic analysis are provided in the Supplementary Material.

## 3 Results and discussion

### 3.1 A colorimetric platform enables ncAA incorporation screening

Ribosomal DNA random mutagenesis coupled with high-throughput screening represents a powerful strategy for identifying beneficial ribosomal modifications that improve the efficiency of GCE. However, efficient selection methods are essential for distinguishing mutations with different activities within large mutational libraries. A previously reported system developed by *Schultz* and colleagues links ncAA incorporation to Gal4-dependent reporter activation in yeast MaV203 [2]. In this system, Gal4 expression is dependent on successful ncAA incorporation, thereby enabling reporter activation only when the target ncAA is incorporated. To minimize background reporter activity and ensure selection specificity, endogenous galactose-responsive transcription factors in *S. cerevisiae* are deleted. However, because reporter activation provides an indirect readout of ncAA incorporation, the resulting signal may not quantitatively reflect the actual incorporation efficiency, particularly under conditions that perturb transcriptional regulation or protein degradation pathways.

To enable direct and visually accessible screening, we constructed a plug-and-play colorimetric reporter in which colony color is coupled to production of an ncAA-dependent enzyme, without requiring genomic manipulation. We selected LacZ because beta-galactosidase converts X-gal into a blue product, allowing colonies with different ncAA incorporation activities to be distinguished on solid medium. Five permissive LacZ residues were individually replaced with TAG codons and expressed from a single-copy plasmid under the *TEF1* promoter and terminator. Structural analysis suggested that these substitutions were unlikely to disrupt LacZ folding or catalytic activity (Fig. S1). We next evaluated these reporters using an evolved *E. coli* TyrRS/tRNATyr CUAsystem for OmeY incorporation [2]. All reporters produced visible β-galactosidase activity upon addition of 1 mM OmeY, but differed in both ncAA-independent background and OmeY-induced colorimetric signal (Fig. 1B). Among them, LacZ(Y96TAG) showed minimal background coloration in the absence of OmeY while maintaining a strong OmeY-dependent signal, providing the most favorable signal-to-background ratio for large-scale mutagenesis screening. Previous studies demonstrated that GFP harboring an amber mutation can serve as a quantitative reporter for ncAA incorporation [11,34]. Consistent with this, a yeast codon-optimized GFP reporter showed markedly increased fluorescence in the presence of 1 mM OmeY compared with ncAA-free conditions (Fig. 1C). We therefore used LacZ(Y96TAG) for primary visual screening and GFP fluorescence for quantitative validation (Fig. 1D).

### 3.2 An 18S rDNA screen identifies ncAA incorporation enhancers

Direct engineering of yeast rDNA is technically difficult because rDNA is present as 100-200 tandem repeats on chromosome XII. To enable tractable manipulation of rDNA, we used a previously engineered yeast strain, HYY34, in which the endogenous rDNA array was deleted and a complete rDNA unit was supplied on a multicopy *URA3* plasmid. The full rDNA cassette was assembled stepwise using Golden Gate cloning (Fig. S2). To construct 18S rDNA mutagenesis libraries, we replaced the 18S rDNA region in the rDNA plasmid with an *RFP* expression cassette. This design enabled visual assessment of library construction: successful insertion of mutant 18S rDNA produced white *E. coli* colonies, whereas empty or incorrectly assembled vectors remained red. Mutant 18S rDNA fragments were generated by error-prone PCR, cloned into the rDNA plasmid library and transformed into yeast for colorimetric screening. The screening workflow is shown in Fig. 1D. Colonies displaying stronger coloration than those carrying wild-type rDNA were selected on solid medium and then re-evaluated under normalized cell density. Candidate 18S rDNA sequences were amplified, sequenced, reconstructed into fresh rDNA plasmids and reintroduced into yeast for colorimetric and GFP-based validation. As candidate strains still retained the wild-type rDNA plasmid, we counterselected this plasmid on 5-FOA medium to generate strains supported exclusively by mutant 18S rDNA.

**Fig. 2:**
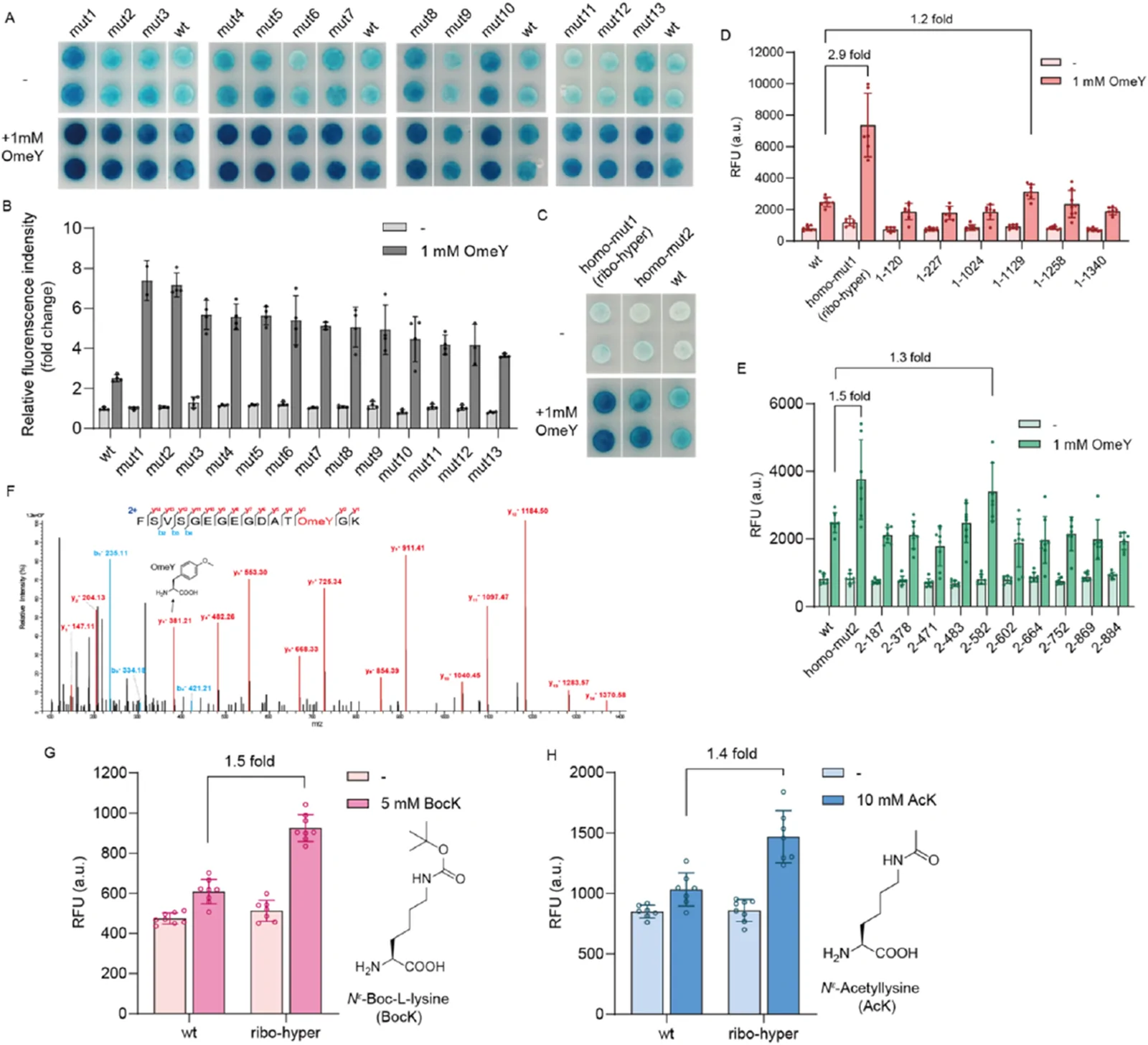
Identification and validation of 18S rDNA mutations that enhance ncAA incorporation. (A) Colorimetric validation of selected 18S rDNA mutants. Mutant strains and the wild-type rDNA strain were compared on the same agar plate. The wild-type rDNA plasmid was retained in the mutant strains. Representative images from two biological replicates are shown. (B) GFP-based quantification of ncAA incorporation in the strains shown in a. Relative GFP production was normalized to that of the wild-type rDNA strain, which was set to 1. The wild-type rDNA plasmid was retained in the mutant strains. Data are from at least two biological replicates. (C) Colorimetric analysis of mut1 and mut2 strains supported exclusively by mutant rDNA after removal of the wild-type rDNA plasmid. (D, E) GFP-based quantification of ncAA incorporation in strains supported exclusively by mut1 or mut2 rDNA. GFP fluorescence was normalized to cell density. Data are presented as mean ± SD from six independent biological replicates. (F) MS/MS fragmentation spectra of tryptic peptides derived from GFP produced in the mut1 strain upon OmeY incorporation. The spectra confirm site-specific OmeY incorporation at the expected TAG-encoded position. (G) Normalized GFP fluorescence in cells expressing CysKRS, a variant of MbPylRS, in the presence of 5 mM BocK. Data are presented as mean ± SD from seven independent biological replicates. (H) Normalized GFP fluorescence in cells expressing PLRS1, a variant of EcLeuRS, in the presence of 10 mM AcK. Data are from at least seven biological replicates.

Based on the initial colorimetric and GFP-based screening, we selected 13 candidate mutants showing increased ncAA-dependent reporter output relative to the wild-type rDNA strain(Fig. 2A and B). Among these candidates, mut1 and mut2 produced the strongest increases in GFP expression. To further validate the contribution of mutant ribosomes, we counterselected the wild-type rDNA plasmid on 5-FOA medium and generated strains supported exclusively by mutant rDNA. In these strains, homo-mut1 showed the strongest OmeY-dependent colorimetric response, whereas homo-mut2 also displayed stronger coloration than the wild-type rDNA strain upon supplementation with 1 mM OmeY (Fig. 2C). These results were consistent with the screening results obtained in strains carrying both mutant and wild-type rDNA plasmids. GFP-based quantification further confirmed enhanced ncAA incorporation. Relative to the wild-type rDNA strain, GFP production increased by 2.9-fold in homo-mut1 and 1.5-fold in homo-mut2 upon OmeY incorporation (Fig. 2D and E). Mass spectrometry verified site-specific incorporation of OmeY at the TAG codon in GFP (Fig. 2F). These results suggested that the colorimetric screening system built in this study could effectively select ncAA incorporation enhancers, and manipulating 18S rRNA exhibited great potential to benefit genetic encoding of ncAAs in yeast.

### 3.3 Cooperative 18S rRNA mutations enhance ncAA incorporation

DNA sequencing revealed that the 18S rDNA of both the mut1 and mut2 variants contained multiple mutations (Table S1). To pinpoint the specific sites governing non-canonical amino acid (ncAA) incorporation efficiency, we generated homozygous strains each harboring a single-site mutation from mut1 or mut2. We specifically targeted sites that disrupted rRNA base-pairing and potentially altered the small ribosomal subunit structure. Notably, the mut1-derived T1129C mutation increased ncAA incorporation by 1.2-fold, whereas no other single mutation from mut1 showed an effect (Fig. 2D and E). Similarly, among the mut2-derived mutations, only T582C produced an approximately 1.3-fold increase. Because no single mutation recapitulated the activity of the full mut1 or mut2 variants, the enhanced phenotype is likely to depend on cooperation among multiple rRNA changes.

To determine whether the enhanced ncAA incorporation observed in the homo-mut1 strain could be generalized across distinct orthogonal translation systems, we evaluated additional ncAA incorporation platforms in the homo-mut1 background. Specifically, we tested CysKRS/tRNA_CUA_ [35], a variant derived from the *Methanosarcina barkeri Mb*PylRS/tRNA_CUA_ pair for *N*^*ε*^-boc-l-lysine (BocK) incorporation, and PLRS/tRNA_CUA_ [36], an engineered *E. coli* LeuRS/tRNA pair for *N*^*ε*^-Acetyllysine (AcK) incorporation. Both systems exhibited enhanced ncAA incorporation in the mut1 strain relative to the wild-type rDNA strain, with GFP production increased by approximately 1.5-fold for CysKRS and 1.4-fold for PLRS (Fig. 2G and H). These results indicate that the enhanced ncAA incorporation phenotype is primarily driven by ribosome engineering and is largely independent of the orthogonal aaRS/tRNA system or the specific ncAA supplied. Given its reproducible activity across systems, we designated the homo-mut1 strain as “ribo-hyper” and used it for subsequent characterization.

### 3.4 The ribo-hyper strain exhibits distinctive translation properties

**Fig. 3:**
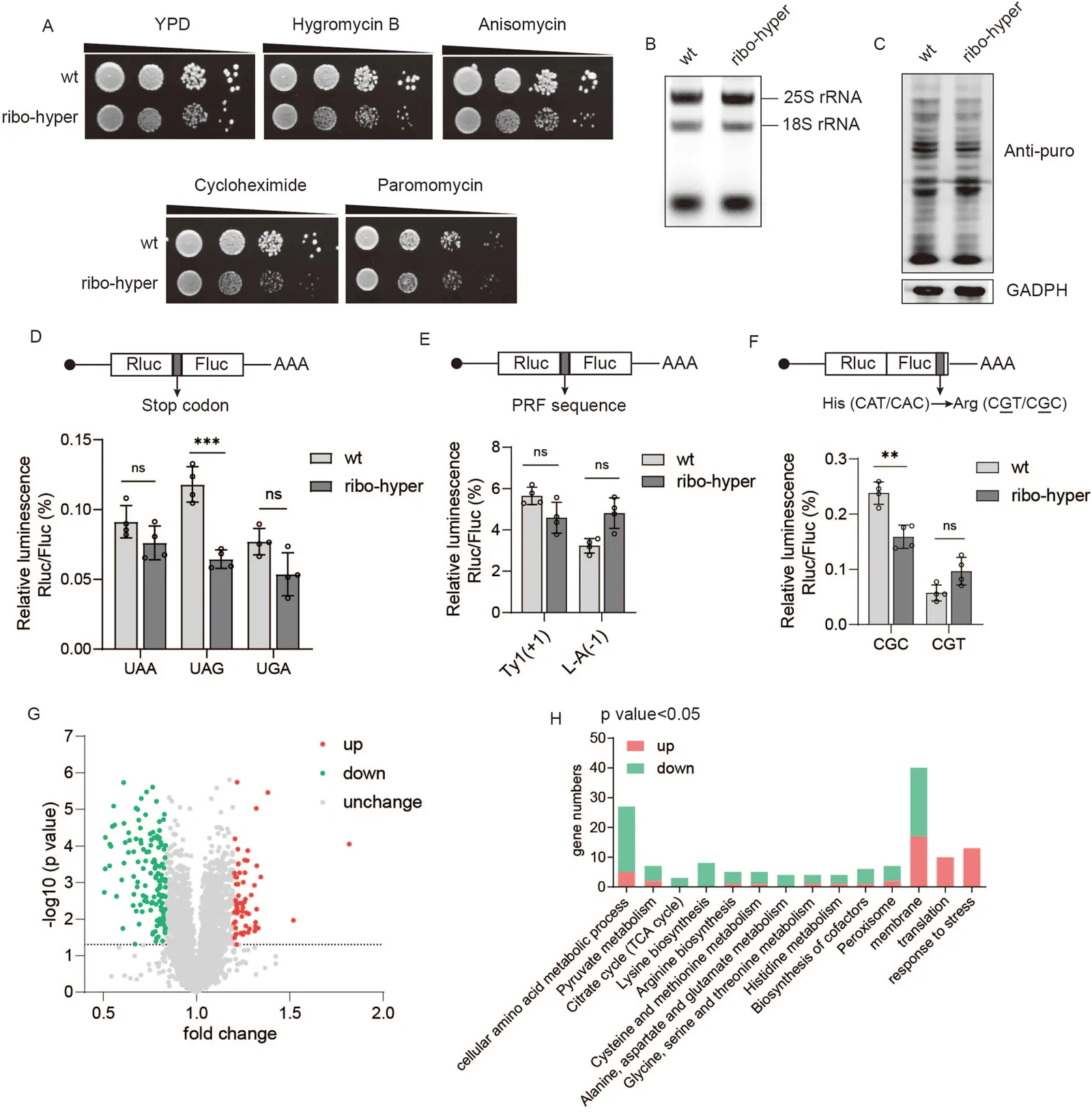
Measurement of translation fidelity and proteomic analysis in the ribo-hyper strain. (A) Serial dilution assays of strains grown for 72 h in the presence of translational inhibitors: 35 μg/ml Hygromycin B; 9 μg/ml Anisomycin; 0.05 μg/ml Cycloheximide and 0.2 mg/ml Paromomycin. (B) Analysis of mature rRNA production. Total RNA was extracted from wild-type and homozygous mut1 strains, and equal amounts of RNA were analyzed by agarose gel electrophoresis. (C) Global translation activity measured by puromycin incorporation. Newly synthesized proteins were labeled with puromycin and detected by immunoblotting using an anti-puromycin antibody. GAPDH served as a loading control. (D) Stop-codon readthrough efficiency measured using reporter constructs containing TAA, TAG or TGA stop codons. (E) Frameshifting efficiency measured using reporter constructs containing −1 or +1 PRF sequence. (F) Amino acid misincorporation measured using Fluc point-mutation reporters in which the His codon in the active site was individually replaced with two Arg codons. The dual-luciferase reporters were constructed according to *Fujii*’s et al. [37]. Values are represented as means ± SD (*t*-test; ***P < 0.001; **P < 0.01; n = 4 independent biological replicates). (G) Volcano plot of protein abundance changes in ribo-hyper relative to the wild-type rDNA strain. The x-axis represents fold change and the y-axis represents -log10(P value) . Proteins with FC > 1.2 or FC < 0.833 and P < 0.05 were considered differentially abundant. Red spot: upregulated proteins, green spot: downregulated proteins, gray spot: unchanged proteins. (H) Protein enrichments from KEGG analysis. Y axis represents the protein numbers, x axis represents the designation of the KEGG pathway. Red column: upregulated proteins, green column: downregulated proteins.

Characterization of the ribo-hyper strain revealed a modest impairment in cellular growth (Fig. 3A). Consistent with the serial-dilution phenotype, liquid-culture growth analysis showed that ribo-hyper exhibited slightly reduced growth relative to the wild-type strain, particularly at later time points (Fig. S3). To examine whether this phenotype was associated with structural or functional changes in the mutant ribosomes, we profiled several translation-related properties. We initially probed ribosomal structural integrity using translation inhibitors, including hygromycin B and cycloheximide (translocation inhibitors), anisomycin (a PTC inhibitor), and paromomycin (a translation fidelity disruptor). The drug sensitivity profiles of ribo-hyper were indistinguishable from those of the wild-type strain (Fig. 3A), suggesting that the 18S rRNA mutations do not cause major defects in the relevant antibiotic-binding sites. Furthermore, agarose gel electrophoresis of total RNA showed no obvious reduction in the mature 25S and 18S rRNA bands in ribo-hyper compared with the wild-type strain (Fig. 3B), arguing against a substantial loss of mature rRNA accumulation. We next assessed global translation activity by puromycin incorporation. The puromycin incorporation assay showed lower puromycin incorporation in ribo-hyper than in the wild-type control, consistent with reduced global translation activity (Fig. 3C). This reduction may contribute to the moderate growth phenotype observed in ribo-hyper.

To examine the translation fidelity of the ribo-hyper strain, we measured translation errors using dual-luciferase reporter constructed according to *Fujii’s* work [37], In these reporters, Renilla luciferase (Rluc) and Firefly luciferase (Fluc) are expressed as a fusion protein, For stop-codon readthrough and frameshifting reporters, individual stop codons or programmed ribosomal frameshifting sequences were inserted between Rluc and Fluc, allowing Fluc activity to be detected only when readthrough or frameshifting occurred. For amino acid misincorporation reporters, the point mutations were introduced to the active site of Fluc, such that Fluc activity could be detected only upon amino acid misincorporation. Translational fidelity was determined by measuring the Fluc/Rluc ratio. We observed that UAG stop codon readthrough was reduced in the ribo-hyper strain compared with the wild-type ribosome, whereas readthrough of UAA and UGA showed no apparent change (Fig. 3D). This result suggests that the 18S rRNA mutations in ribo-hyper selectively enhance termination at the amber codon, leading to more efficient translational termination at the amber codon than that observed with the wild-type ribosome. Frameshifting frequencies were also comparable between the ribo-hyper strain and the wild-type ribosome (Fig. 3E), indicating that the 18S rRNA mutations did not broadly affect programmed ribosomal frameshifting. By contrast, amino acid misincorporation at the CGC codon was reduced in the ribo-hyper strain (Fig. 3F), indicating improved decoding accuracy. Given that high-fidelity ribosomes have been reported to promote ncAA incorporation efficiency [38], we propose that the 18S rRNA mutations in ribo-hyper facilitate more accurate codon-anticodon recognition, thereby enhancing efficient ncAA incorporation.

To assess the global effects of the 18S rRNA mutation in the ribo-hyper strain, we performed proteomic analysis. Several amino acid biosynthetic pathways were prominently affected, with proteins involved in lysine and arginine biosynthesis showing reduced abundance. However, supplementation with lysine and arginine did not restore the growth of ribo-hyper (Fig. S4), suggesting that the growth phenotype is unlikely to result solely from limitation of these two amino acids. Supplementation with lysine and arginine resulted in a modest increase in OmeY-dependent GFP production in both the wild-type and ribo-hyper backgrounds, while the enhanced ncAA-dependent reporter output of ribo-hyper was maintained (Fig. S5). Together with the altered abundance of translation- and stress-response-associated proteins, these observations suggest that the growth phenotype may reflect broader changes in cellular homeostasis. In addition, proteins associated with translation and stress resistance were upregulated, consistent with compromised ribosome function and activation of cellular stress responses caused by the 18S rRNA mutation.

### 3.5 Translation-associated quality-control pathways influence ncAA incorporation

**Fig. 4:**
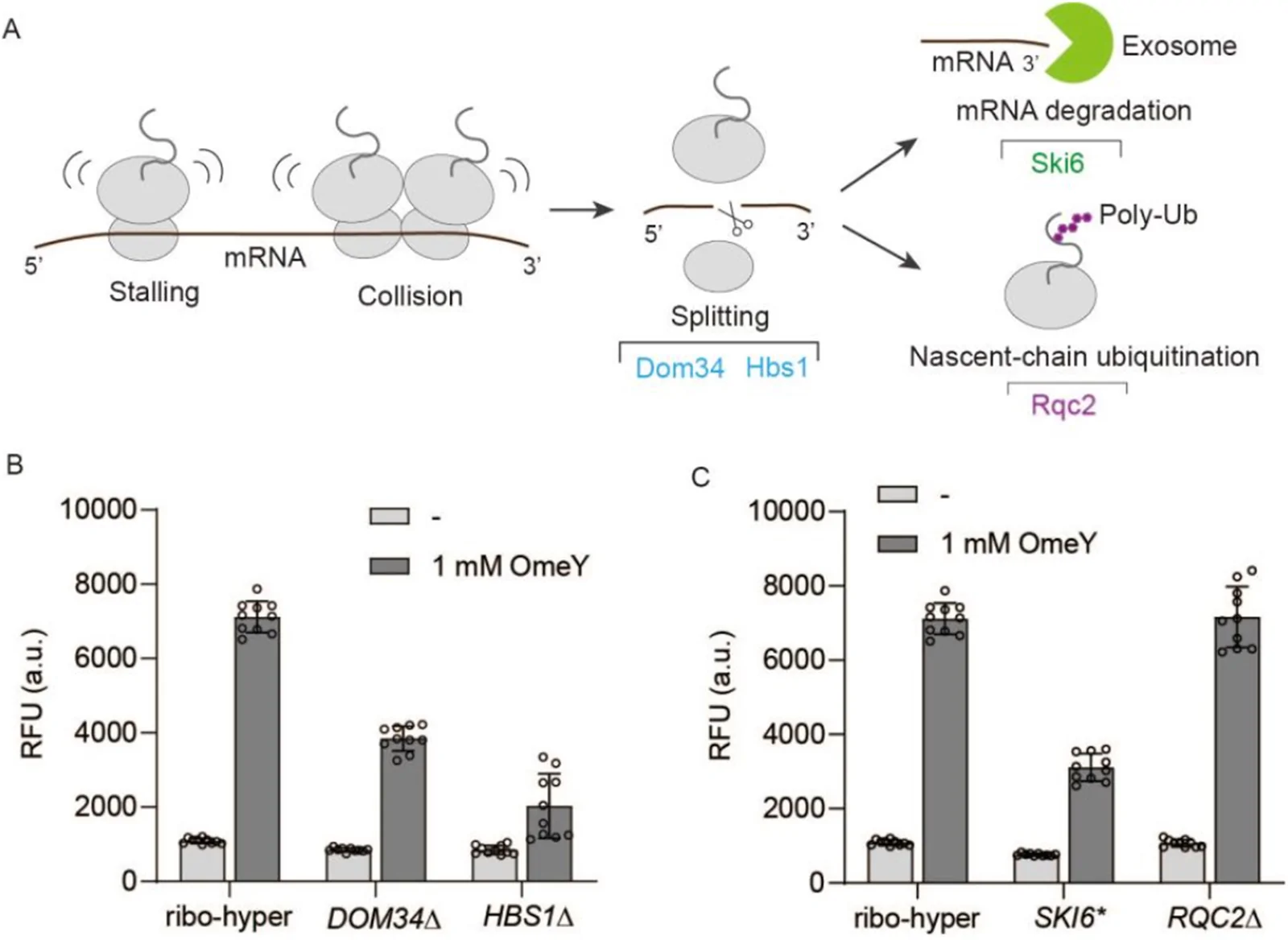
Effects of translation quality-control pathways on ncAA incorporation. (A) Schematic of ribosome-rescue processes activated upon ribosome stalling. (B, C) ncAA incorporation efficiency following perturbation of selected translation quality-control factors. GFP fluorescence was normalized to cell density. Data are presented as mean ± SD from ten independent biological replicates.

Cells have evolved surveillance pathways that monitor multiple steps of translation to preserve fidelity. Aberrant translation can activate mRNA and ribosome quality-control pathways, leading to degradation of engaged transcripts, clearance of incomplete nascent peptides and splitting of ribosomal subunits [11,27]. Because GCE repurposes stop codons for ncAA decoding, quality-control pathways beyond nonsense-mediated decay may be engaged during ncAA-containing peptide synthesis and thereby affect incorporation efficiency. To determine whether translation quality-control pathways affect ncAA incorporation in the ribo-hyper strain, we perturbed key factors involved in the downstream resolution of stalled ribosomes (Fig. 4A). Under unfavorable translational conditions, ribosomes can stall on problematic mRNAs. The Dom34-Hbs1 complex promotes dissociation of stalled ribosomes, facilitates ribosome recycling and can stimulate downstream endonucleolytic cleavage of the associated mRNA [29]. Deletion of *DOM34* or *HBS1* markedly reduced ncAA-containing protein production in the ribo-hyper strain (Fig. 4B), suggesting that efficient ribosome rescue and recycling contribute to productive ncAA incorporation. Following ribosome dissociation, stalled translation complexes can be resolved through additional downstream processes. One such process is degradation of the exposed mRNA by the RNA exosome. Because *SKI6*, which encodes a core exosome component involved in 3′-5′ RNA processing and degradation, is essential in yeast, we introduced previously reported mutations that impair exosome exonucleolytic activity while maintaining cell viability [39]. These mutations reduced ncAA incorporation efficiency, indicating that exosome-mediated mRNA turnover may help maintain efficient production of ncAA-containing proteins. A second downstream pathway involves rescue of an aberrant 60S subunit that remains attached to an obstructing nascent-chain-tRNA conjugate. This process is initiated by the ribosome quality-control factor Rqc2, which senses problematic 60S complexes and promotes recruitment of ubiquitin ligases for nascent-chain degradation [40]. We found that deletion of *RQC2* had little effect on ncAA-containing protein production (Fig. 4C), suggesting Rqc2 is not important for ncAA incorporation under the conditions tested. These findings suggest that ribosome rescue and coupled mRNA degradation are more important for maintaining ncAA incorporation efficiency in the ribo-hyper strain than Rqc2-dependent nascent-chain quality control.

## 4 Conclusions

This study establishes a yeast ribosome-engineering strategy for improving eukaryotic genetic code expansion. By coupling an ncAA-dependent LacZ reporter to visual colony screening and using GFP fluorescence for quantitative validation, we identified 18S rRNA variants that enhance ncAA-containing protein production. The strongest variant, ribo-hyper, improved OmeY-dependent GFP production by 2.9-fold and also enhanced incorporation mediated by additional orthogonal aaRS/tRNA systems. These results support the conclusion that the eukaryotic ribosome itself can be tuned to improve GCE in vivo.

A central feature of the screening strategy is that reporter output is directly linked to production of an ncAA-containing enzyme. This design avoids the need for extensive genomic rewiring of host transcriptional circuits and makes the system compatible with routine laboratory screening. The LacZ readout is especially useful for primary screening because it allows activity differences to be recognized visually across large libraries, whereas the GFP reporter provides a quantitative secondary assay. This two-step design reduces the chance that candidates are selected solely on the basis of colony size or background reporter activation.

Beyond 18S rRNA engineering, the LacZ(TAG)-based colorimetric platform could potentially be adapted to screen other components of the translational machinery. Because this study demonstrates that modification of the small-subunit rRNA can enhance ncAA-dependent protein production, other ribosomal components, including ribosomal proteins, may also represent useful engineering targets. Similarly, translation-associated factors such as release factors and elongation factors, as well as orthogonal tRNAs and aminoacyl-tRNA synthetases, could be subjected to library screening using the same ncAA-dependent reporter framework. Thus, the platform may provide a broadly adaptable strategy for engineering multiple layers of the translation machinery to improve genetic code expansion.

The mutational analysis suggests that the ribo-hyper phenotype depends on the combined effects of multiple 18S rRNA mutations. Individual mutations from mut1 or mut2 produced only modest improvements, whereas the complete variants showed substantially stronger activity. Structural mapping of the ribo-hyper mutations onto the *Saccharomyces cerevisiae* 80S ribosome revealed that the mutated residues are spatially distributed across the 18S rRNA rather than clustered around the decoding center (Fig. S6). The stronger phenotype of the complete variant is therefore consistent with a collective, context-dependent effect of multiple rRNA substitutions. These dispersed mutations may collectively influence small-subunit conformational dynamics or functional-state equilibria and thereby indirectly modulate decoding. However, the present structural mapping does not establish the precise molecular mechanism, and further structural and biophysical analyses will be required to define how these mutations alter ribosome function.

The improved ncAA-dependent protein production in ribo-hyper was accompanied by reduced global translation activity, indicating that bulk translational capacity and productive decoding of a reassigned codon can be decoupled. A moderate reduction in translational throughput may alter ribosomal dynamics and increase the time window available for productive accommodation of the ncAA-charged suppressor tRNA at UAG. In addition, the reduced endogenous UAG readthrough and amino acid misincorporation observed in ribo-hyper are consistent with increased codon-anticodon discrimination, which may favor recognition of the cognate suppressor tRNA. The 18S rRNA mutations may also alter ribosome-tRNA or ribosome-translation-factor interactions in a manner that improves functional accommodation of the orthogonal tRNA. Together, these effects may enhance ncAA decoding despite reduced global protein synthesis, although direct kinetic and structural studies will be required to define the underlying mechanism.

Our analysis of translation-associated quality-control pathways suggests that ribosome rescue and mRNA turnover can influence ncAA-containing protein output. Deletion of *DOM34* or *HBS1* reduced ncAA incorporation in ribo-hyper, as did mutations that impair exosome-associated RNA degradation. By contrast, deletion of *RQC2* had little effect under the conditions tested. These results argue that efficient recycling of stalled ribosomes and associated mRNA processing may support sustained ncAA-containing protein synthesis, whereas Rqc2-dependent nascent-chain quality control is not a major limiting factor in this assay. However, these pathways are interconnected, and genetic perturbations may affect mRNA abundance or ribosome availability indirectly. Future work measuring reporter mRNA levels, ribosome occupancy and nascent-chain stability will be needed to define the precise step at which each pathway acts.

In summary, this work provides a practical framework for engineering the eukaryotic ribosome to enhance GCE and identifies ribosome-associated quality-control processes as additional determinants of ncAA-containing protein production. The ribo-hyper strain should provide a useful platform for improving ncAA incorporation in yeast, while the screening strategy can be expanded to explore larger rRNA libraries and additional ribosomal regions. More broadly, the results highlight translation homeostasis as a tunable layer for improving eukaryotic synthetic biology.

## CRediT authorship contribution statement

**Xiaoxu Chen:** Writing – original draft, Methodology, Funding acquisition, Data curation, Conceptualization. **Wenlu Shen:** Investigation, Data curation. **Xianqing Chen:** Software, Investigation. **Fangfang Zhang:** Investigation, Data curation. **Bo Yang:** Software, Resources. **Zhiyong Yue:** Writing – review & editing, Conceptualization. **Guanghou Zhao:** Writing – review & editing, Supervision.

## Declaration of competing interest

The authors declare the following financial interests/personal relationships which may be considered as potential competing interests: Wenlu Shen is currently employed by Biocytogen (Beijing) Co., Ltd.
