# Supplementary material for: Ribosome engineering enhances genetic code expansion in Saccharomyces cerevisiae
Source: Synth Syst Biotechnol. 2026 Aug 29;18:76–84. doi: 10.1016/j.synbio.2026.08.009 (PMC13545395; doi:10.1016/j.synbio.2026.08.009)
Supplement: Supplementary file 1 [file mmc1.docx]

**Supplementary Material**

**Ribosome Engineering Enhances Genetic Code Expansion in *Saccharomyces cerevisiae***

**Contents**

Supplementary Methods: TMT-based quantitative proteomic analysis................................... 1
Fig. S1. Structural localization of LacZ residues selected for TAG substitution.......................2
Fig. S2. Stepwise Golden Gate assembly of the rDNA expression plasmid............................2
Fig. S3. Liquid-culture growth of wild-type and ribo-hyper strains...........................................3
Fig. S4. Lysine/arginine supplementation and ribo-hyper growth............................................3
Fig. S5. Lysine/arginine supplementation and OmeY-dependent GFP production..................4
Fig. S6. Structural mapping of ribo-hyper 18S rRNA mutations...............................................4
Table S1. 18S rDNA mutations identified in screening.............................................................4
Table S2. Strains used in this study..........................................................................................5
Table S3. Plasmids used in this study.......................................................................................6
Table S4. Primers used in this study.........................................................................................7

**Supplementary Methods: TMT-based quantitative proteomic analysis**

Protein samples were extracted using SDT buffer and quantified using the BCA method. Proteins were digested with trypsin using the filter-aided sample preparation (FASP) procedure, and the resulting peptides were desalted using C18 cartridges. For quantitative proteomic analysis, 100 μg of peptides from each sample were labeled with tandem mass tag (TMT) reagents according to the manufacturer’s instructions (Thermo Scientific). Equal amounts of labeled peptides were pooled and fractionated using a High-pH Reversed-Phase Peptide Fractionation Kit (Thermo Scientific).

Peptide fractions were separated using an Easy-nLC system. Mobile phase A consisted of 0.1% formic acid in water, whereas mobile phase B consisted of 84% acetonitrile containing 0.1% formic acid. Peptides were loaded onto an Acclaim PepMap100 C18 trap column (100 μm×2 cm; Thermo Scientific) and separated using an EASY C18 analytical column (10 cm×75 μm inner diameter, 3 μm particles; Thermo Scientific) at a flow rate of 300 nL/min.

LC-MS/MS analysis was performed using a Q-Exactive mass spectrometer operated in positive-ion and data-dependent acquisition mode. Full MS scans were acquired over an m/z range of 300–1800 at a resolution of 70,000 at m/z 200, with an automatic gain control target of 1 × 10^6 and a maximum injection time of 50 ms. Dynamic exclusion was set to 60 s. Following each full scan, the 20 most abundant precursor ions were selected for HCD fragmentation. The isolation window was 2 m/z, MS/MS spectra were acquired at a resolution of 17,500 at m/z 200, and the normalized collision energy was 30 eV.

Raw MS data were processed using MASCOT and Proteome Discoverer and searched against the UniProtKB Saccharomyces cerevisiae protein database (uniprot_Saccharomyces_cerevisiae_53530_20240422.fasta). Trypsin was specified as the proteolytic enzyme with up to two missed cleavages. Carbamidomethylation of cysteine and TMT modification of peptide N-termini and lysine residues were specified as fixed modifications, whereas oxidation of methionine was treated as a variable modification. Precursor and fragment mass tolerances were set to ±20 ppm and 0.1 Da, respectively. A decoy-database strategy was used, and peptide identifications were filtered at an FDR of ≤1%.

Protein abundance ratios were calculated as the median of the ratios of unique peptides assigned to each protein. Peptide ratios were normalized using the median protein ratio, such that the median protein ratio after normalization was 1. Proteins were considered quantifiable when intensity values were available in more than half of the biological replicates in at least one comparison group. No additional missing-value imputation was introduced during the revised differential-abundance analysis. Differential protein abundance between ribo-hyper and the wild-type strain was assessed using a t-test based on three independent biological replicates. Proteins with a fold change (FC) >1.2 or <0.833 and a P value <0.05 were considered differentially abundant. The FC threshold was used as an effect-size filter to exclude very small abundance fluctuations and to focus the analysis on proteins showing a measurable change in abundance.

Fig. S1. Structural localization of the five LacZ residues selected for TAG substitution. The selected residues are shown in orange on the three-dimensional LacZ structure, which was visualized using PyMOL.

Fig. S2. Stepwise Golden Gate assembly strategy for constructing the rDNA expression plasmid. Individual genetic modules were first assembled into first-level intermediate constructs, each containing three to four genes. Two first-level intermediates were then combined to generate a second-level intermediate, followed by final assembly of the complete rDNA expression plasmid.

Fig. S3. Liquid-culture growth of the wild-type and ribo-hyper strains. Cells were cultured in YPD medium, and OD600 was measured at the indicated time points. Data are presented as mean ± SD from three independent biological replicates.

Fig. S4. Effect of lysine and arginine supplementation on the growth of ribo-hyper. The ribo-hyper strain was cultured in YPD medium with or without supplementation of 1 mM lysine and 1 mM arginine (L+A), and OD600 was measured at the indicated time points. Data are presented as mean ± SD from three independent biological replicates.

Fig. S5. Effect of lysine and arginine supplementation on OmeY-dependent GFP production. GFP fluorescence was measured in wild-type and ribo-hyper strains in the absence or presence of 1 mM OmeY, with or without supplementation of 1 mM lysine and 1 mM arginine (L+A). Data are presented as mean±SD from three independent biological replicates.

Fig. S6. Structural mapping of the 18S rRNA mutations in ribo-hyper. The mutation sites identified in ribo-hyper were mapped onto the structure of the *S. cerevisiae* 80S ribosome ([PDB 6TNU]). The 18S rRNA is shown in blue, the mutated residues are highlighted as red spheres, and the tRNAs are shown in orange and yellow. The mapped mutations are spatially dispersed across the 18S rRNA rather than concentrated near the decoding center.

Table S1. 18S rDNA mutations identified in strains with improved ncAA incorporation.

| Strains | Mutation numbers | Mutation sites |
| --- | --- | --- |
| mut1(ribo-hyper) | 10 | T120C, T227C, A421G, T753C, C897T, T1024C, T1098C, T1129C, T1258C, T1340C |
| mut2 | 10 | G187A, A378G, A471C, A483G, T582C, T602C, T664C, A752G, A869C, A884G |
| mut3 | 4 | T29C, T64C, T293C, A328G |
| mut4 | 14 | A85G, T319C, A542G, T781G, T830C, T947C, T1056C, A1139G, T1247C, A1375G, T1558C, T1585C, A1633G, A1678G |
| mut5 | 1 | A140G |
| mut6 | 1 | A221T |
| mut7 | 8 | A51G, T174C, C543T, T759C, G871A, T1120C, G1271A, G1685A |
| mut8 | 2 | C1284T, T1704C |
| mut9 | 3 | G63A, C1173T, G1273A |
| mut10 | 4 | G876T, C1220T, C1599T, T1738C |
| mut11 | 4 | A28G, T110C, T785C, T1608C |
| mut12 | 2 | C424T, A1436G |
| mut13 | 10 | T73C, T260C, T626C, T785C, A1137G, A1411C, T1585C, C1596T, G1668T, T1738C |

Table S2. Strains used in this study.

| Name | Genotype | Description |
| --- | --- | --- |
| BY4741 | *his3*Δ1 *leu2*Δ0 *met15*Δ0 *ura3*Δ0 | Lab stored |
| HYY34 | BY4741*, rdn*Δ::*NAT* pRDNwt-U | Lab stored |
| CXY0 | BY4741*,* rdnΔ::NAT pRDNwt-L | This study |
| CXY1 | BY4741*, rdn*Δ::NAT pRDNmut1-L | This study |
| CXY2 | BY4741*, rdn*Δ::NAT pRDNmut2-L | This study |
| CXY3 | BY4741*, rdn*Δ::NAT pRDNmut1-L *HEL2*Δ | This study |
| CXY4 | BY4741*, rdn*Δ::NAT pRDNmut1-L *LTN1*Δ | This study |
| CXY5 | BY4741*, rdn*Δ::NAT pRDNmut1-L *RQC2*Δ | This study |
| CXY6 | BY4741, *rdn*Δ::NAT pRDNmut1-L *SKI6*(K62E,S63D,R95D,R96D) | This study |
| CXY7 | BY4741*, rdn*Δ::NAT pRDNmut1-L *DOM34*Δ | This study |
| CXY8 | BY4741*, rdn*Δ::NAT pRDNmut1-L *HBS1*Δ | This study |

Table S3. Plasmids used in this study.

| Name | Description |
| --- | --- |
| pRDNwt-U | 2μ plasmid carrying *RDN-WT rDNA URA3 amp^r^* |
| pRDNrfp-L | 2μ plasmid carrying *RDN-RFP rDNA LEU2 amp^r^* |
| pRDNwt-L | 2μ plasmid carrying *RDN-WT rDNA LEU2 amp^r^* |
| pRDNmut1-L | 2μ plasmid carrying *RDN-MUT1 rDNA LEU2 amp^r^* |
| pRDNmut2-L | 2μ plasmid carrying *RDN-MUT2 rDNA LEU2 amp^r^* |
| pEcOmeYRS-GFP | 2μ plasmid carrying evolved *EcTyrRS/tRNA* pair, p*TEF1*-*yeGFP*(TAG)-t*CYC1* *TRP1 amp^r^* |
| pMbpylRS-4tRNA-GFP | 2μ plasmid carrying evolved *MbpylRS* and 4 copy of *tRNA*, p*TEF1*-*yeGFP*(TAG)-t*CYC1* *TRP1 amp^r^* |
| pEcPLRS-GFP | 2μ plasmid carrying evolved *EcLeuRS/tRNA* pair, p*TEF1*-*yeGFP*(TAG)-t*CYC1* *TRP1 amp^r^* |
| pCX-LacZ96 | CEN plasmid carring p*TEF1-LacZ*96TAG-t*TEF1* *MET17 amp^r^* |
| pCX-gRNA(HEL2) | 2μ plasmid carrying p*tRNA*(*Tyr*)-HDV ribozyme-*HEL2* gRNA-sgRNA-t*SRN52 HIS3 amp^r^* |
| pCX-gRNA(LTN1) | 2μ plasmid carrying p*tRNA*(*Tyr*)-HDV ribozyme-*LTN1* gRNA-sgRNA-t*SRN52 HIS3 amp^r^* |
| pCX-gRNA(RQC2) | 2μ plasmid carrying p*tRNA*(*Tyr*)-HDV ribozyme-*RQC2* gRNA-sgRNA-t*SRN52 HIS3 amp^r^* |
| pCX-gRNA(SKI6) | 2μ plasmid carrying p*tRNA*(*Tyr*)-HDV ribozyme-*SKI6* gRNA-sgRNA-t*SRN52 HIS3 amp^r^* |
| pCX-gRNA(DOM34) | 2μ plasmid carrying p*tRNA*(*Tyr*)-HDV ribozyme-*DOM34* gRNA-sgRNA-t*SRN52 HIS3 amp^r^* |
| pCX-gRNA(HBS1) | 2μ plasmid carrying p*tRNA*(*Tyr*)-HDV ribozyme-*HBS1* gRNA-sgRNA-t*SRN52 HIS3 amp^r^* |

Table S4. Primers used in this study.

| Name | Sequence | Description |
| --- | --- | --- |
| 18S-BsmbI-F | CTAGTGcgtctcgTAGTTATCTGGTTGATCCTGCCAGTAG | For 18S rDNA amplification |
| 18S-BsmbI-R | CTAGTGcgtctcgTAGTTATCTGGTTGATCCTGCCAGTAG |  |
| 18S-SQ-1 | GCCCAAAGTTCAACTACG | For 18S rDNA sequencing |
| 18S-SQ-2 | CCTTAACGAGGAACAATTGG |  |
| 18S-SQ-3 | GCCTGCGGCTTAATTTGA |  |
| pTEF-F | agcgagtcagtgagcgaggaCACAATGCATACTTTGTACGTTC | For yeGFP insertion into plasmids |
| tTER-R | TTGCGTATTGGGCGCTCTTCCGCTTGGCGATTAAGTTGGGTAACG |  |
| tRNA-SalI-F | ctagtgGTCGACATCGATGTGTGTTAtatgtacctc | For pMbpylRS-4tRNA plasmid construction |
| tRNA-PstI-R | ctagtgCTGCAGccgCTCGAGaagcattaatgaagaataactacaggAG |  |
| pTEF-BamHI-F | GAAAATACCGCATCAGGCGCGgatccCACACACCATAGCTTCAAAATG |  |
| pTEF-SpeI-R | GCGGCCGCTCTAGAACTAGTTTTGTAATTAAAACTTAGATTAGATTGCTATGC | For pCX-LaZ96 plasmids construction |
| tTEF-NotI-F | actagttctagagcggccgcGGAGATTGATAAGACTTTTCTAGTTGC |  |
| tTEF-R | CTAAAGGGAACAAAAGCTGGAGCTCCTGAAAAAAGAGGGGAATTTTTAGATAC |  |
| LacZ-F | TCTAAGTTTTAATTACAAAACTAGTATGACCATGATTACGGATTCACTG |  |
| LacZ-1 | TGACCATGATTACGGATTCACTGGCCGTCGTTTTAC |  |
| LacZ-2 | CAGGGTTTTCCCAGTCACGACGTTGTAAAACGACGGCCAGTGAATCCGTAATCATGGTC |  |
| LacZ-3 | CGTGACTGGGAAAACCCTGGCGTTACCCAACTTAATCGCCTTGCAGCACATCCCCCTTT |  |
| LacZ-4 | GGCGATCGGTGCGGGCCTCTTCGCTATTACGCCAGCTGGCGAAAGGGGGATGTGCTGCA |  |
| LacZ-5 | AGGCCCGCACCGATCGCC |  |
| Y96-R | TTGGTGTAGATGGGCGCATCcTAACCGTGCATCTGCCAG |  |
| Y96-F | GATGCGCCCATCTACACCAA |  |
| F114-R | CGGATTCTCCGTGGGAACctaCGGCGGATTGACCGTAATG |  |
| F114-F | GTTCCCACGGAGAATCCG |  |
| Y254-R | GCCATAAAGAAACTGTTACCCGTAGCTAGTCACGCAACTCGCCG |  |
| Y254-F | CTACGGGTAACAGTTTCTTTATGGC |  |
| F385-R | CGAACAGCGCACGGCGTTctaGTTGTTCTGCTTCATCAGCAG |  |
| F385-F | AACGCCGTGCGCTGTTCG |  |
| Y579-R | CGGGTTGCCGTTTTCATCCTATTTAATCAGCGACTGATCCACC |  |
| Y579-F | GATGAAAACGGCAACCCG |  |
| LacZ-R | CTAGTGGGTCTCATACGTTTTTGACACCAGACCAACTG |  |
| flag-F | CTAGTGGGTCTCTCGTACGCTGCAGGTCGAC |  |
| flag-R | CTAGTGGCGGCCGCTTAATGGTGATGGTGGTGATG |  |
| gRNA-HEL2-F | cttaCGGCTCCATCTAGAGTGATC | For gRNA plasmids construction |
| gRNA-HEL2-R | aaacGATCACTCTAGATGGAGCCG |  |
| gRNA-LTN1-F | cttaGCAGCCATTAATTAGCTCCA |  |
| gRNA-LTN1-R | aaacTGGAGCTAATTAATGGCTGC |  |
| gRNA-RQC2-F | cttaGCTTGCATCAAAGTGTTCGG |  |
| gRNA-RQC2-R | aaacCCGAACACTTTGATGCAAGC |  |
| gRNA-SKI6-F | cttaAGATACAAACCTCCCTGGTG |  |
| gRNA-SKI6-R | aaacCACCAGGGAGGTTTGTATCT |  |
| gRNA-DOM34-F | CTTaGTATAAATCAGACACCGCGG |  |
| gRNA-DOM34-R | AAACCCGCGGTGTCTGATTTATAC |  |
| gRNA-HBS1-F | CTTAGCGCAGTTACAGGATTACCA |  |
| gRNA-HBS1-R | AAACTGGTAATCCTGTAACTGCGC |  |
| HEL2-donor-1-F | GGAAGACTTGTAATTAAGCATTATCTCG | For amplification of donor fragments |
| HEL2-donor-1-R | CCTAATTCTTGAAAAGAAGTATAGCCAC |  |
| HEL2-donor-2-F | ACTTCTTTTCAAGAATTAGGGTTGGAAATATATTTCTAACCTGTAACTGACAATAG |  |
| HEL2-donor-2-R | GTCAACCACAGGAAATTCTGG |  |
| LTN1-donor-1-F | CCTTAGCATGGGAGAGGTCTC |  |
| LTN1-donor-1-R | TAAGAACCAACTATTGCTTGAACTTTTTTTG |  |
| LTN1-donor-2-F | CAAGCAATAGTTGGTTCTTAACTATCGCATATAAATTTCATATAAATGTACTAC |  |
| LTN1-donor-2-R | GGGGACATTCAATAGAATTTCATCAATGG |  |
| RQC2-donor-1-F | CCTCAACTTAGCATGTGCTGATTACAC |  |
| RQC2-donor-1-R | CGCTATTAATCTCTTGCCTGCC |  |
| RQC2-donor-2-F | CAGGCAAGAGATTAATAGCGATCATATGAGATGAAAAGAAAATAGACAGC |  |
| RQC2-donor-2-R | GCATCCAGAGTTTCTAAACCACC |  |
| SKI6-donor-1-F | CCAAGATTGCAAGACCAAATGGATACCTCAAAGGC |  |
| SKI6-donor1-R | AAACATTCTCACCAGGGAGGTTTGTATCTCAAGAACTTGTTGTTCATTCTTGTGGCTTG |  |
| SKI6-donor-2-F | CCTCCCTGGTGAGAATGTTTGAGAAGAATG |  |
| SKI6-donor-2-R | CGTTCTCTAACCTATCTAACGG |  |
| DOM34-UP-F | CTCATCTCATCGCTTCCC |  |
| DOM34-UP-R | ATTTTTGCTATGATAGGAATGCGATAATTG |  |
| DOM34-down-F | CAATTATCGCATTCCTATCATAGCAAAAATGATGGTGAGGAGTAGATTTACTATGTAAG |  |
| DOM34-down-R | AGGCGCCTAAACGCAGAT |  |
| HBS1-UP-F | CCAGAGAGGACCGTAAGC |  |
| HBS1-UP-R | CTCGATAGTCAATAGTTGTCGAAGC |  |
| HBS1-down-F | GCTTCGACAACTATTGACTATCGAGAAGGGTGAAAATAGATGATAAGAATTTTATACGC |  |
| HBS1-down-R | TTCATCTATTGAATCCATAATAACTGTCTG |  |
